# Present and Future Perspectives in the Treatment of Liver Fibrosis

**DOI:** 10.3390/ph18091321

**Published:** 2025-09-03

**Authors:** Lucia Cerrito, Linda Galasso, Jacopo Iaccarino, Alessandro Pizzi, Fabrizio Termite, Giorgio Esposto, Raffaele Borriello, Maria Elena Ainora, Antonio Gasbarrini, Maria Assunta Zocco

**Affiliations:** 1 Department of Internal Medicine and Gastroenterology, Fondazione Policlinico Universitario Agostino Gemelli IRCCS, Catholic University of Rome, 00168 Rome, Italy; lucia.cerrito@policlinicogemelli.it (L.C.); jacopo.iaccarino01@icatt.it (J.I.);; 2 CEMAD Digestive Disease Center, Fondazione Policlinico Universitario Agostino Gemelli IRCCS, Catholic University of Rome, 00168 Rome, Italy

**Keywords:** liver fibrosis, hepatic stellate cells (HSC), transforming growth factor-β (TGF-β), farnesoid X receptor (FXR) agonists, gut–liver axis

## Abstract

Background/Objectives: Liver fibrosis is a progressive consequence of chronic liver injury that can evolve into cirrhosis, liver failure, or hepatocellular carcinoma, representing a major global health burden. Fibrogenesis is driven by hepatic stellate cell (HSC) activation, excessive extracellular matrix deposition, and structural disruption of liver tissue, with transforming growth factor-β (TGF-β) signaling and inflammatory mediators as central pathways. Current therapies primarily target the underlying causes, which may halt disease progression but rarely reverse established fibrosis. This review aims to outline current and emerging therapeutic strategies for liver fibrosis, informing both clinical practice and future research directions. Methods: A narrative synthesis of preclinical and clinical evidence was conducted, focusing on pharmacological interventions, microbiota-directed strategies, and innovative modalities under investigation for antifibrotic activity. Results: Bile acids, including ursodeoxycholic acid and derivatives, modulate HSC activity and autophagy. Farnesoid X receptor (FXR) agonists, such as obeticholic acid, reduce fibrosis but are limited by adverse effects. Fatty acid synthase inhibitors, exemplified by denifanstat, show promise in metabolic dysfunction-associated steatohepatitis (MASH). Additional strategies include renin–angiotensin system inhibitors, omega-3 fatty acids, and agents targeting the gut–liver axis. Microbiota-directed interventions—probiotics, prebiotics, symbiotics, antibiotics (e.g., rifaximin), and fecal microbiota transplantation—are emerging as potential modulators of barrier integrity, inflammation, and fibrogenesis, though larger clinical trials are required. Reliable non-invasive biomarkers and innovative trial designs, including adaptive platforms, are essential to improve patient selection and efficiently evaluate multiple agents and combinations. Conclusions: Novel modalities such as immunotherapy, gene editing, and multi-targeted therapies hold additional potential for fibrosis reversal. Continued translational efforts are critical to establish safe, effective, and accessible treatments for patients with liver fibrosis.

## 1. Introduction

Hepatic fibrosis is a pathological condition characterized by excessive extracellular matrix (ECM) deposition in response to chronic liver injury, leading to progressive scarring of the hepatic parenchyma [1,2]. Over time, fibrosis may progress to cirrhosis, liver failure, and hepatocellular carcinoma (HCC), making it a key determinant of chronic liver disease outcomes [2,3,4,5,6]. The etiologies of hepatic fibrosis are diverse and include metabolic, infectious, autoimmune, toxic, and genetic conditions. Globally and in Europe, the leading causes are excessive alcohol consumption, chronic viral hepatitis, particularly hepatitis B virus (HBV) and hepatitis C virus (HCV), and metabolic dysfunction-associated steatotic liver disease (MASLD) [7,8,9]. Alcohol-related injury involves oxidative stress and lipid dysregulation [8,9,10], whereas viral hepatitis promotes fibrosis through immune-mediated mechanisms, with coinfections (e.g., HBV/HDV or HCV) accelerating progression toward cirrhosis and HCC [11,12,13,14,15,16,17]. MASLD encompasses a spectrum from simple steatosis to metabolic-associated steatohepatitis (MASH), with fibrosis progression influenced by individual wound-healing responses [18,19]. Less common but clinically relevant contributors include inherited metabolic disorders such as Wilson’s disease and hemochromatosis [20,21,22,23], as well as autoimmune liver diseases including autoimmune hepatitis (AIH), primary biliary cholangitis (PBC), and primary sclerosing cholangitis (PSC) [24,25,26,27].

Given the substantial global burden and the limitations of current therapies, there is a strong rationale for developing innovative antifibrotic strategies targeting both disease etiology and fibrogenic pathways.

The aim of this review is to provide a comprehensive and updated overview of the molecular mechanisms underlying hepatic fibrosis, while also exploring current and emerging therapeutic strategies. Particular attention is given to novel drug classes targeting key fibrogenic pathways, the role of the gut–liver axis, and the integration of non-invasive diagnostic methods. We also discuss challenges in treatment efficacy, long-term safety, and the need for personalized and etiology-specific approaches to liver fibrosis management.

## 2. Methods

The literature search for this narrative review was conducted using the PubMed, Scopus, Web of Science, and Embase databases. Relevant studies published up to April 2025 were identified using a combination of keywords and Medical Subject Headings (MeSH) related to liver fibrosis and its pathogenesis, including but not limited to, the following: liver fibrosis, fibrogenesis, epigenetics, immune response, microbiota, antifibrotic therapy, non-invasive biomarkers, and clinical trials. Studies were included if they provided original data or comprehensive reviews on the pathogenesis, diagnosis, or treatment of liver fibrosis in humans. Preclinical studies were included when relevant to mechanistic insights or therapeutic targets. Articles were selected based on their relevance, originality, and contribution to the current understanding of liver fibrosis and its therapeutic management, with priority given to peer-reviewed articles, meta-analyses, and recent clinical trials. Given the narrative (non-systematic) nature of this review, a formal quality scoring or risk-of-bias assessment was not applied.

## 3. Molecular Mechanisms Underlying Liver Fibrosis

### 3.1. Genetic Mechanisms of Liver Fibrosis

Several genetic factors contribute to the susceptibility and progression of liver fibrosis, a condition characterized by excessive extracellular matrix (ECM) deposition [28]. Among the most influential is the patatin-like phospholipase domain-containing 3 (PNPLA3) gene on chromosome 22 [29,30,31]. The rs738409 C>G variant (I148M) reduces PNPLA3 enzymatic activity, disrupting lipid hydrolysis and promoting hepatocellular injury. This triggers activation of Kupffer cells (KCs) and hepatic stellate cells (HSCs), with sustained HSC activation driving ECM overproduction and fibrotic remodeling [30,32,33]. Another key gene is transmembrane 6 superfamily member 2 (TM6SF2) on chromosome 19, which regulates hepatic lipid metabolism [34,35]. The rs58542926 C>T variant (E167K) impairs TM6SF2 function in very-low-density lipoprotein (VLDL) secretion, leading to intracellular lipid accumulation, hepatic steatosis, and increased susceptibility to lipotoxicity, oxidative stress, and inflammation—all central to fibrogenesis [36,37,38,39,40]. This variant may also contribute to a pro-fibrotic hepatic microenvironment, facilitating progression from chronic liver disease to hepatocellular carcinoma (HCC) [41,42].

### 3.2. Epigenetic Mechanisms of Liver Fibrosis

Epigenetic mechanisms modulate gene expression without altering the DNA sequence and are central to the development and progression of liver fibrosis [43]. Altered DNA methylation is commonly observed in fibrotic liver tissue. Hypermethylation of anti-fibrotic genes such as PPARγ and SOCS1 reduces protective pathways that regulate inflammation and fibrosis resolution [44,45,46,47,48]. Conversely, hypomethylation of pro-fibrotic genes like TGFβ1 promotes sustained fibrogenic signaling, leading to continuous HSC activation and excessive ECM production [49,50,51].

Several epigenetic regulators influence HSC transdifferentiation and fibrosis. EZH2, part of the Polycomb repressive complex, promotes histone methylation and gene silencing, accelerating fibrotic progression [52,53,54]. HDACs and SIRT1 modulate histone acetylation to control the expression of fibrosis-related genes [55,56,57], while KDM6B removes repressive histone marks such as H3K27me3, enabling transcription of pro-fibrotic genes [58].

Non-coding RNAs also play key roles. MicroRNAs (miR-21, miR-29, miR-122) regulate HSC activation, ECM deposition, and the transition from fibrosis to HCC [59,60,61,62]. Long non-coding RNAs (H19, MEG3, MALAT1) modulate chromatin structure and transcriptional networks, influencing pro-fibrotic gene expression, HSC activation, and matrix remodeling [63,64,65,66]. Together, these epigenetic mechanisms and non-coding RNAs orchestrate fibrogenic pathways, and their dysregulation contributes critically to liver fibrosis progression.

Table 1 summarizes the impact of genetic and epigenetic mechanisms on fibrosis.

### 3.3. Hepatic Stellate Cells

HSCs are fundamental mediators in the onset and progression of liver fibrosis, acting as the principal source of ECM components during chronic liver injury. Their involvement in fibrogenesis has been clearly demonstrated in experimental models, where selective depletion of HSCs significantly attenuates fibrotic responses induced by agents such as carbon tetrachloride (CCl_4_) or procedures like bile duct ligation (BDL) [67,68]. In healthy liver tissue, HSCs reside in the perisinusoidal space of Disse, where they remain quiescent and function primarily as storage units for vitamin A-rich lipid droplets. However, in the context of chronic liver injury, HSCs undergo a profound phenotypic transformation into activated myofibroblast-like cells. The activation of HSCs is a key step in the development of liver fibrosis and occurs in response to various pro-fibrogenic stimuli generated during chronic liver injury. The main triggers for HSC activation can be categorized into cellular, soluble, metabolic, oxidative, and mechanical stimuli [50].

#### 3.3.1. Cellular and Immune Stimuli for HSC Activation

During chronic liver injury, hepatocyte death, whether via apoptosis, necrosis, or necroptosis, triggers the release of damage-associated molecular patterns (DAMPs), including HMGB1, heat shock proteins, and extracellular matrix components. These DAMPs activate resident Kupffer cells (KCs) and recruit circulating monocytes/macrophages to the site of injury [69]. Activated immune cells secrete a range of pro-inflammatory and pro-fibrogenic mediators, such as tumor necrosis factor alpha (TNF-α), interleukin-1 beta (IL-1β), TGF-β1, and platelet-derived growth factor (PDGF), establishing a microenvironment that both amplifies inflammation and drives HSC activation [70,71]. TGF-β1 is a central fibrogenic cytokine acting through the canonical SMAD pathway, where SMAD2 and SMAD3 are phosphorylated, complex with SMAD4, and translocate to the nucleus to induce transcription of extracellular matrix (ECM) genes including COL1A1, COL3A1, and TIMP1 [72,73,74]. TGF-β1 also engages non-canonical pathways, including MAPK (ERK, JNK, p38), PI3K/AKT, and Rho-like GTPases, further promoting HSC activation and survival. Chronic viral hepatitis exacerbates this process, as hepatocyte apoptosis and persistent inflammation drive sustained TGF-β1 release [48]. PDGF, particularly the PDGF-BB isoform, regulates HSC proliferation and ECM production through PDGFR-β signaling, activating downstream PI3K/AKT, MAPK/ERK, and JAK/STAT cascades. PDGF is produced by KCs, sinusoidal endothelial cells, platelets, and injured hepatocytes, forming paracrine loops that reinforce fibrogenesis [75,76,77,78]. Hepatic macrophages, comprising resident KCs and infiltrating monocyte-derived macrophages, modulate fibrosis. Classically activated M1 macrophages, induced by IFN-γ, secrete TNF-α, IL-1β, and IL-6, enhancing inflammation and promoting HSC transdifferentiation into myofibroblasts. Alternatively activated M2 macrophages, stimulated by IL-4 and IL-13, release TGF-β1 and PDGF, supporting HSC survival and ECM deposition, thereby facilitating tissue remodeling and fibrosis progression [79,80,81]. Natural killer (NK) cells have a context-dependent role. During early injury, they exert antifibrotic effects by inducing HSC apoptosis via perforin, granzyme B, and TRAIL, and by producing IFN-γ, which suppresses HSC activation and ECM synthesis. In chronic or advanced fibrosis, NK cell cytotoxicity is impaired due to elevated TGF-β levels and inhibitory receptor expression (e.g., NKG2A), leading to a dysfunctional phenotype that may indirectly sustain fibrosis through profibrotic cytokine secretion and interactions with other immune cells [82,83].

#### 3.3.2. Oxidative Stress and HSC Activation

In alcohol-induced liver disease, ethanol metabolism generates reactive oxygen species (ROSs) and acetaldehyde, which cause hepatocellular damage and activate Kupffer cells (KCs), amplifying the inflammatory response that drives HSC transdifferentiation [84,85]. Oxidative stress is a key driver of hepatic fibrogenesis—arising from ROS produced by injured hepatocytes—activated KCs, infiltrating neutrophils, and NADPH oxidase (NOX) isoforms, particularly NOX1 and NOX4 in HSCs [86]. ROS-induced DNA damage activates signaling pathways such as p53, NF-κB, and JNK, enhancing transcription of fibrogenic genes including COL1A1, TIMP1, and ACTA2 (α-smooth muscle actin) [87,88]. ROSs also compromise mitochondrial integrity, leading to dysfunction and activation of the intrinsic apoptotic pathway via cytochrome c release and caspase cascade activation, resulting in hepatocyte apoptosis [89]. The resulting apoptotic bodies and cellular debris act as DAMPs, recognized by pattern recognition receptors (PRRs) on HSCs and immune cells. Among these, Toll-like receptor 4 (TLR4) is particularly important in fibrosis progression. TLR4 is upregulated in activated HSCs and responds to DAMPs or gut-derived lipopolysaccharide (LPS), activating the MyD88-dependent pathway and NF-κB-mediated transcription of pro-inflammatory and pro-fibrotic cytokines [90]. TLR4 signaling also downregulates Bambi, a pseudoreceptor that normally inhibits TGF-β signaling [91,92]. This crosstalk establishes a feed-forward loop that reinforces HSC activation, ECM deposition, and fibrotic remodeling.

#### 3.3.3. Metabolic Stimuli for HSC Activation

In MASLD and its progressive inflammatory form, MASH, metabolic dysregulation plays a central role in hepatic fibrosis. Accumulation of free fatty acids (FFAs) and lipotoxic intermediates, including ceramides, diacylglycerols (DAGs), and oxidized lipids, induces hepatocyte damage, mitochondrial dysfunction, endoplasmic reticulum (ER) stress, and oxidative injury, leading to the release of damage-associated molecular patterns (DAMPs) [93,94]. These signals activate Kupffer cells (KCs), infiltrating macrophages, and hepatic stellate cells (HSCs), initiating a fibrogenic cascade.

Lipotoxic species also act directly on HSCs via pattern recognition receptors (TLRs and PRRs), triggering intracellular signaling through NF-κB, JNK, and p38 MAPK. These pathways converge on the transcription of pro-fibrotic genes such as ACTA2 (α-SMA), COL1A1, and TGF-β1, promoting HSC transdifferentiation into myofibroblast-like cells capable of synthesizing extracellular matrix (ECM) [95].

HSC activation is accompanied by profound metabolic reprogramming. Activated HSCs shift toward aerobic glycolysis, with upregulation of enzymes including hexokinase 2 (HK2), pyruvate kinase M2 (PKM2), and lactate dehydrogenase A (LDHA), supporting energy production and biosynthesis of nucleotides, amino acids, and lipids required for ECM synthesis, proliferation, and survival. Pharmacological inhibition of glycolysis reduces HSC activation and collagen production in experimental models [96,97].

Concomitantly, glutaminolysis fuels the tricarboxylic acid (TCA) cycle, sustains anabolic processes, and maintains redox balance via NADPH production. The enzyme glutaminase (GLS1) is upregulated in activated HSCs, and its inhibition decreases fibrotic gene expression and HSC viability [98,99].

#### 3.3.4. Mechanical and ECM-Driven Activation of HSCs in Liver Fibrosis

In liver fibrosis, the progressive accumulation and cross-linking of ECM components, particularly collagen types I and III, result in increased tissue stiffness, which profoundly alters the hepatic microenvironment. HSCs, the primary effector cells in fibrogenesis, sense these mechanical changes via integrin-mediated mechanotransduction, triggering the activation of downstream signaling pathways such as RhoA/ROCK and the Hippo-YAP/TAZ axis [100,101]. Under physiological conditions, YAP and TAZ, the main transcriptional coactivators of the Hippo pathway, are tightly regulated through phosphorylation by upstream kinases MST1/2 and LATS1/2. This phosphorylation promotes their cytoplasmic retention and subsequent proteasomal degradation, preventing aberrant gene transcription [102,103]. However, in the fibrotic liver, YAP/TAZ become aberrantly activated in HSCs, translocate to the nucleus, and interact with TEAD transcription factors to induce the expression of genes involved in ECM production, cell proliferation, and survival [104]. This transcriptional program contributes to excessive matrix deposition, progressive tissue stiffening, and architectural distortion of the liver parenchyma.

In parallel, the Wnt/β-catenin signaling pathway has also emerged as a crucial regulator of hepatic fibrogenesis. Aberrant activation of Wnt ligands and stabilization of β-catenin in HSCs promote their transdifferentiation into myofibroblasts, enhance ECM production, and sustain proliferation and survival of activated HSCs. Furthermore, crosstalk between Wnt/β-catenin and other profibrotic pathways, including TGF-β and YAP/TAZ, amplifies fibrogenic gene expression and contributes to the persistence of liver injury [105,106].

Simultaneously, the RhoA/ROCK signaling cascade plays a central role in promoting the fibrogenic phenotype of HSCs. ROCK activation enhances the formation of actin stress fibers and upregulates fibrogenic markers such as α-smooth muscle actin (α-SMA) and collagen type I [107,108].

Notably, RhoA/ROCK signaling is functionally intertwined with other profibrotic pathways, including TGF-β and Hippo-YAP/TAZ. ROCK-induced cytoskeletal tension facilitates both the mechanical activation of latent TGF-β and the nuclear translocation of YAP/TAZ, creating a feed-forward loop that amplifies fibrogenic gene expression and sustains the fibrotic response. Together, these interconnected signaling networks orchestrate the pathological remodeling that underlies liver fibrosis [77].

#### 3.3.5. Gut Microbiota and HSC Activation

The relationship between gut microbiota and liver fibrosis is an expanding field of research, focusing on the gut-liver axis, which allows gut-derived products to reach the liver via the portal vein [109,110]. Gut microbiota plays a fundamental role in the development of liver fibrosis whatever the cause of liver damage, but with specific microbial hallmarks according to the different etiologies (viral, MASLD, ALD, cholestatic disease) [111,112,113,114,115]. The “gut–liver axis” has a key role in the genesis and evolution of liver fibrosis, mainly related to the unbalance between the increase in noxious anaerobes (*Proteobacteria* and *Bacilli*) and the reduction in beneficial anaerobes (*Clostridia*). While the latter help maintain the health and integrity of the intestinal wall, the former can damage the intestinal barrier. This disruption may allow bacteria and their inflammatory products to translocate into the portal bloodstream. Once they reach the liver, these components contribute to the development of a dysregulated and unfavorable hepatic microenvironment. This process includes the release of pro-inflammatory and fibrogenic cytokines, aberrant recruitment and activation of immune cells, and abnormal stimulation of intrahepatic T cell receptors [116,117].

In cases of dysbiosis, the altered microbiota generates harmful substances such as LPS, ethanol, and bile acid metabolites that trigger hepatic inflammation and activate HCSs, promoting fibrosis [118,119]. Central to this interaction is TLR4, which is expressed on various liver cells, including HSCs and sinusoidal endothelial cells [120]. When LPS, derived from Gram-Negative bacteria like *Escherichia coli* (*Enterobacteriaceae*), binds to TLR4, it initiates a signaling cascade through MyD88, which activates IRAK kinase, leading to the activation of the transcription factor NF-κB. NF-κB activation results in the production of pro-inflammatory cytokines, including TNF-α, IL-1β, and IL-6, fostering an inflammatory environment that further activates HSCs and drives fibrosis [121,122]. Additionally, dysbiosis disrupts bile acid metabolism, influencing the signaling pathways of farnesoid X receptor (FXR) and TGR5, which are essential in regulating liver function and inflammation [123,124,125,126,127].

Certain microbiota species are closely associated with liver fibrosis. Enterobacteriaceae, such as *Escherichia coli*, produce LPS that activates Kupffer cells and induces the release of inflammatory cytokines, directly contributing to fibrosis. *Streptococcus* spp. has been linked to more advanced stages of fibrosis in MASLD [128], while *Veillonella* spp., often enriched in cirrhosis, is associated with increased lactate and ammonia levels, exacerbating liver dysfunction [129]. In contrast, beneficial species such as *Faecalibacterium prausnitzii* produce butyrate, which has anti-inflammatory effects and helps maintain the integrity of the gut barrier [130]. *Akkermansia muciniphila* enhances gut barrier function and is inversely related to liver disease severity, offering protective benefits [131]. Members of the *Ruminococcaceae* family, producers of short-chain fatty acids (SCFAs), also protect against inflammation, while *Bifidobacterium* spp. support the gut barrier and reduce endotoxin translocation. Together, these molecular pathways highlight how dysbiosis can drive liver fibrosis through inflammatory, immune, and metabolic disturbances [132,133,134]. Understanding these microbial signatures presents promising opportunities for non-invasive biomarkers, therapeutic interventions, and potential treatments, such as probiotics or fecal microbiota transplantation, in managing liver fibrosis.

**Figure 1:**
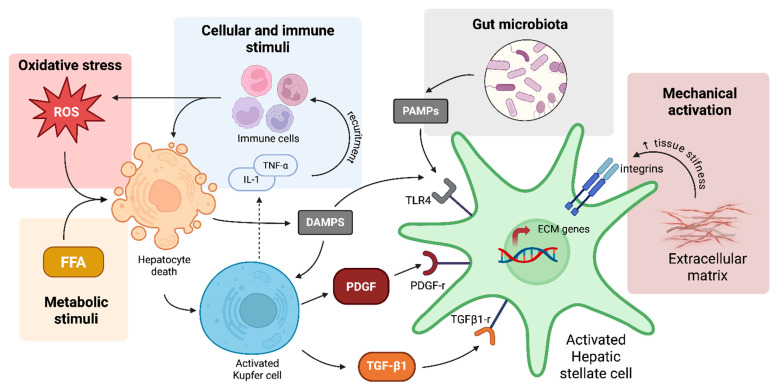
Schematic representation of the main cellular and molecular mechanisms involved in liver fibrosis. Chronic liver injury activates hepatocytes and Kupffer cells, which release inflammatory and fibrogenic mediators such as TNF-α, IL-1β, and TGF-β1. These factors induce hepatic stellate cell activation and extracellular matrix deposition. Signals from platelet-derived growth factor (PDGF), oxidative stress, and gut-derived endotoxins such as lipopolysaccharide (LPS) further promote fibrogenesis. Mechanotransduction pathways including Hippo/YAP/TAZ and RhoA/ROCK are also involved in maintaining the activated state of stellate cells in response to matrix stiffness. Components of the gut–liver axis, particularly microbiota-derived signals, contribute to immune dysregulation and hepatic inflammation.

Figure 1 summarizes all the mechanisms mentioned above that underlie hepatic fibrosis.

## 4. Fibrosis Quantification: Available Methodologies

Accurate quantification of liver fibrosis is essential for diagnosis, staging, and monitoring of chronic liver diseases. Histological evaluation via liver biopsy remains the reference standard, with staging systems such as METAVIR [135,136]. However, due to invasiveness, potential complications, and sampling variability, non-invasive alternatives are increasingly preferred. Imaging-based techniques are widely used in clinical practice. Transient elastography (TE, FibroScan) measures liver stiffness in kilopascals (kPa) through shear wave velocity. Stiffness values correlate with fibrosis stage: >7–8 kPa indicates significant fibrosis (≥F2), and >12.5–14 kPa suggests cirrhosis (F4), although thresholds vary by etiology [137,138,139,140]. TE is rapid, reproducible, and operator-independent but may be less accurate in patients with obesity, ascites, or acute inflammation. Shear wave elastography (SWE) offers real-time elastographic maps integrated with standard ultrasound, performing reliably in patients with high BMI or ascites [141,142,143]. Both TE and SWE provide reproducible and clinically actionable measurements, suitable for fibrosis screening, longitudinal follow-up, and treatment planning. Magnetic resonance elastography (MRE) provides spatially resolved liver stiffness measurements with superior accuracy, particularly for early fibrosis and heterogeneous patterns [144,145]. Guidelines increasingly support a combined approach, integrating non-invasive elastography with biochemical markers (e.g., APRI, FIB-4, FAST) and clinical data to stratify patients and reduce biopsy requirements, enabling more precise, patient-tailored management [146,147,148,149].

## 5. Therapeutic Approaches for Liver Fibrosis

Regardless of its multiple etiologies, in each of them liver fibrosis has a common pathogenesis, starting from damage to hepatocytes and cholangiocytes, subsequent dysregulation of the inflammatory processes, and abnormal healing mechanisms that lead to an accumulation of fibrosis and ECM components [2].

Regarding the treatment of etiologies, during the last decades the revolutionary introduction of antiviral drugs for the treatment of chronic viral infections (HBV and HCV), drastically reduced the number of people that face a progressive development of fibrosis due to these pathologies [150,151]. Unlike what happened decades ago, the precocious detection and consequent treatment of inherited and autoimmune liver disorders grant the possibility to avoid the development of fibrosis or cirrhosis [20,23,25,26,27]. A similar reasoning can be made for alcoholic liver disease, in which the elimination of the etiological agent allows the mechanisms that generate liver damage to be turned off [152].

During the last years, an intense focus has been placed on both diagnosing and treating fibrosis in MASLD, which has become the most prevalent among the etiologies of chronic liver disease [153]. Basically, lifestyle modification (regular diet, caloric restriction and improvement of physical activity) remain the principal therapeutic tools, due to preliminary evidence of its impact on weight loss and the subsequent effect on liver damage: the reduction of liver fat is achieved by a loss of 5% of weight, while the improvement of liver inflammation is observed after the loss of 7–10% of body weight and the reduction of liver fibrosis is determined by the loss of more than 10% of weight. Despite these encouraging data, the potential role of weight loss strategy in the reduction of liver fibrosis still needs further evidence [154]. Kleiner et al. highlighted the impact of lifestyle modification on liver fibrosis in a cohort of 446 adults with MASLD/MASH, who underwent long-term follow-up (average 4.9 years) and periodic liver biopsy in order to verify the evolution of liver fibrosis: in this way, they highlighted that the improvement of disease activity led to fibrosis regression, while the worsening of MASLD was associated with fibrosis progression. 39.1% patients presented a reduction of 1 or more stages in liver fibrosis compared to baseline, associated with a lower baseline insulin levels (20 μU/mL versus 33 μU/mL, *p* = 0.02) and more relevant decrease in alanine aminotransferase and aspartate aminotransferase values [155].

There are numerous studies that testify the pharmacological efforts made to obtain a valid weapon in the treatment of liver fibrosis, due to their potential positive implications [156].

### 5.1. Liver-Directed Thyroid Hormone Receptor Agonists (THR)

These is growing interest in thyroid hormone receptor agonists (THR), selective for b subtype of the receptor of thyroid hormone, as a possible therapeutic solution for MASH. In fact, these patients are often affected also by hypothyroidism, which is related to worse outcomes in terms of liver inflammation and fibrosis degree, due to the effect of thyroid hormones on the inhibition of lipogenesis in the liver [157] and of TGF-β (transforming growth factor β1) pathway [158].

Resmetirom is an oral agonist of the receptor β for the thyroid hormone that has been approved in the United States of America in 2024 due to the encouraging results from the MAESTRO-NASH trial, a phase III trial in non-cirrhotic MASH patients with stage 2–3 fibrosis. This molecule provided better results compared to placebo in the reduction in inflammation and in the reduction in fibrosis progression [159]. In fact, patients with stage 2 fibrosis had a slower progression, with good tolerability and safety profile, since the reported adverse events mainly regarded nausea (22%), diarrhea (33%), vomiting (11%) [2]. Since this trial is continuing and updated results are expected, it is still to be established if this drug can bring further benefits, especially in terms of preventing the development of cirrhosis, and regarding the confirmation of long-term safety [160,161].

For these reasons, the drug has been approved only in the U.S., and it can be prescribed to subjects with F2–F3 fibrosis (but not cirrhotic patients), detected through liver biopsy or non-invasive methods. An important clinical consideration concerns the optimal strategy for combining resmetirom with glucagon-like peptide-1 receptor agonists (GLP1RAs) in patients who are already receiving the latter as part of their treatment for type 2 diabetes mellitus, as was the case for 14% of participants in the study by Harrison et al. [160].

### 5.2. Incretin Mimetics

Incretin mimetics, also known as glucagon-like peptide-1 receptor agonists (GLP-1 RAs), are a class of drugs that mimic the action of endogenous incretin hormones. Beyond glycemic regulation, incretin pathways also influence appetite control, gastric emptying, and energy balance, making them attractive targets for the treatment of metabolic diseases. Semaglutide, liraglutide and tirzepatide are GLP1RA that have been approved for the treatment of type 2 diabetes and obesity due to their impact on weight loss and appetite reduction, with important positive effects on renal and cardiovascular functions [162,163]. In a phase 2 trial, Newsome et al. found no statistically significant difference between semaglutide and placebo in terms of liver fibrosis improvement: 43% of patients receiving 0.4 mg semaglutide achieved ≥1-stage fibrosis improvement without worsening of NASH, compared to 33% in the placebo group (*p* = 0.48) [164]. These results, though numerically favorable to semaglutide, were not statistically significant, raising early concerns about its efficacy in fibrosis reversal. However, in the subsequent phase 3 ESSENCE trial, higher-dose semaglutide (2.4 mg) showed improved outcomes: after 72 weeks of treatment, MASH resolution occurred in 62.9% of patients versus 34.1% on placebo, and fibrosis improvement was observed in 37% of patients receiving semaglutide compared to 22.5% with placebo. Differences in dosing, study design, and statistical power between the two trials may partly explain the divergent outcomes. Moreover, semaglutide was associated with a higher incidence of these events compared to placebo [165].

A phase 2, randomized, double-blind trial evaluating the effect of semaglutide with combination regimens of cilofexor (farnesoid X receptor agonist) and firsocostat (acetyl-coenzyme A carboxylase inhibitor) on patients with MASLD-related liver cirrhosis (F4 fibrosis) is still ongoing [166].

Another phase 2b trial by Loomba et al. involving 392 patients with F3–4 MASLD-related fibrosis, examined the effect of cilofexor, firsocostat, selonsertib (inhibitor of Apoptosis Signal-regulating Kinase 1-ASK1) or placebo, alone or in combinations of two drugs, administered once per day for 48 weeks and noticed that cilofexor/firsocostat had a potential effect on fibrosis regression in subjects with advanced MASLD-related fibrosis, with a significant reduction (*p* = 0.040) in the score of the Machine Learning NASH Clinical Research Network (ML NASH CRN) and a passage from F3–F4 to a value equal or inferior to F2 in the histological samples [167]. Selonsertib alone was administered for 48 weeks by Harrison et al. in a phase III trial involving patients with F3–4 fibrosis but did not prove significant results as a potential antifibrotic molecule [168].

Tirzepatide is a GLP-1 (glucagon-like peptide 1) and GIP (glucose-dependent insulinotropic peptide) dual agonist; its role in the treatment of liver fibrosis besides the resolution of MASH was hinted by a phase 2, dose-finding, multicenter, double-blind, randomized trial by Loomba et al. who documented an improvement of at least one stage of fibrosis in 55% of patients undergoing treatment with 5 mg of tirzepatide, 51% with 10 mg, and with 15 mg of tirzepatide, versus 30% with placebo. Adverse events were predominantly gastrointestinal and dose-related [169].

Nahra et al. highlighted the potential role of cotadutide (dual GLP-1 and glucagon receptor agonist) in the reduction in fibrosis scores in patients with MASLD (*p* = 0.010), while liraglutide did not have the same outcomes regarding liver fibrosis, measured with fibrosis-4 (FIB-4) index and “non-alcoholic fatty liver disease” NAFLD fibrosis score (NFS) [170]. Glucagon-like peptide-1 receptor agonists are generally well tolerated, though gastrointestinal side effects—including nausea, vomiting, and decreased appetite—are commonly reported.

Survodutide is a subcutaneous dual agonist of glucagon receptor and GLP-1 receptor that proved superiority compared to placebo in a phase 2 trial by Sanyal et al., who documented MASH improvement as primary endpoint, without fibrosis worsening. The fibrosis reduction of at least one stage was achieved in 34% patients of the survodutide 2.4 mg group, 36% with 4.8 mg, 34% with 6.0 mg, and 22% with placebo [171]. However, since fibrosis regression was a secondary endpoint and the study was not powered for that outcome, these findings should be interpreted cautiously pending phase 3 confirmation.

### 5.3. Inhibitors of Sodium–Glucose Cotransporter 2 (SGLT2)

The inhibitors of sodium–glucose cotransporter 2 (SGLT2), officially approved for the treatment of type 2 diabetes (dapagliflozin, licogliflozin, empagliflozin), are also applied in the treatment of patients with heart failure and chronic kidney disease [158,172]. Their in vitro effect in the regulation of microRNAs involved in the activation of HSCs lead several researchers to deepen their understanding on their potential involvement in the amelioration of liver fibrosis, particularly in MASLD [173]. A small study by Shimizu et al. noticed that patients treated with dapagliflozin could undergo a reduction in liver fibrosis (but only in those with significant grade of fibrosis), even if the researchers could not exclude that loss in body weight and in visceral adipose tissue determined by dapagliflozin could be related with the reduction in both liver steatosis and fibrosis [174]. In the research by Pradhan et al., based on large cohorts of patients with type-2 diabetes in the United Kingdom, the authors confronted separately GLP-1RAs and sodium–glucose cotransporter 2 (SGLT-2) inhibitors with dipeptidyl peptidase 4 (DPP-4) inhibitors. They found that the subjects treated with GLP-1RAs, compared with those undergoing DPP-4 inhibitors, did not have a lower risk of developing liver cirrhosis, while patients treated with SGLT-2 inhibitors had a lower risk of liver cirrhosis compared to those undergoing DPP-4 inhibitors [175].

### 5.4. Peroxisome Proliferator-Activated Receptor Agonists

The group of peroxisome proliferator-activated receptor (PPAR) is a family of nuclear receptor transcription factors with several functions: among the three different isoforms (PPAR-a, PPAR-d or PPAR-b and PPAR-ɣ), PPAR-a is the most highly represented in the liver [158,176]; the deregulation of PPAR activity is involved in the alteration of lipid metabolism, enhances inflammation, and enhances fibrogenesis [177]. Preliminary studies suggested that the activation of PPAR is able to reduce mild fibrosis and even lead to an amelioration of extrahepatic complications [178].

Boyer-Diaz et al. elaborated preclinical model of rats with decompensated cirrhosis and liver cells from subjects with advanced chronic liver disease (ACLD), achieving promising results administering lanifibranor, a pan-PPAR agonist (at dosage 100 mg/kg/day), versus placebo on liver fibrosis and portal hypertension. In fact, the rats undergoing treatment with lanifibranor had a reduction in portal pressure of 15% (*p* = 0.003) compared to placebo with an improvement in liver vascular resistance and no reduction in the effective blood flow. The practical demonstration of this result in the rats treated with lanifibranor was the reduction in ascites and liver inflammation, regression of fibrosis (−32%, *p* = 0.020), improvement in liver microvascular activity and of the phenotype of both sinusoidal endothelial cells and HSCs. These findings are encouraging but they derive from a controlled rodent model that, despite its mechanistic value, may not fully reflect the complexity and heterogeneity of human cirrhosis. However, similar effects were also observed ex vivo in liver cells derived from cirrhotic human tissue, supporting biological plausibility [179]. Moreover, in a clinical setting, Francque et al. highlighted the role of lanifibranor in the reduction of at least one stage of liver fibrosis in patients with MASH (without the worsening of liver baseline disease), measured through “Steatosis Activity Fibrosis” (SAF) scoring system (including scores of ballooning and inflammation): 48% with 1200 mg lanifibranor, 34% with 800 mg, 29% in the placebo group; the risk ratio was 1.7 in the first group compared to placebo (95% CI, 1.2 to 2.5) and it was 1.2 in the second group versus placebo (95% CI, 0.7 to 1.9). Better results were also documented in the group of patients with advanced fibrosis. However, treatment was associated with weight gain and peripheral edema, particularly in patients receiving the higher dose of 1200 mg. Despite the rigor of this trial, it should be noted that the study population included patients with MASH but not decompensated cirrhosis, and the follow-up was limited to histological endpoints [180].

### 5.5. Metformin

Metformin is a first-line glucose-lowering medication with adjunctive anti-inflammatory and anti-fibrotic results, particularly in patients affected by type 2 diabetes, that are clinically associated with MASLD and liver fibrosis in up to 20% subjects [156,181,182]. It is a valuable molecule that has been investigated for its potential role in both treatment and prevention of liver fibrosis through the inhibition of several pathways involved in fibrogenesis. However, most evidence to date derive from animal models or small observational studies, and its direct impact on fibrosis regression in humans remains uncertain. Larger, controlled clinical trials are required to determine whether its anti-inflammatory and metabolic effects can be translated into consistent antifibrotic benefits [183].

Lee et al. observed that more than 50% patients undergoing treatment with metformin presented a regression of liver fibrosis [184], while other researchers demonstrated that metformin inhibited the expression of TGF-β, enhanced the phosphorylation of SMAD3 [185] and counteracted the activation of HSCs [186]. Other potentially valuable effects of this drug are the possibility to increase transplant-free survival in patients with MASLD, type-2 diabetes, and biopsy-proven F3–F4 fibrosis (but without effects on liver decompensation) [187] and the reduction in the risk of primary hepatic cancer and extrahepatic tumors [188,189].

### 5.6. Fibroblast Growth Factor 21 (FGF21) Analogues

Fibroblast Growth Factor 21 (FGF21) is a hormone-like growth factor produced mainly in adipose tissue and liver, playing a fundamental role in the regulation of the metabolism of both glucose and lipids. It reduces lipogenesis and increases hepatic sensitivity to insulin. It is involved in specific metabolic diseases such as type 2 diabetes, contributing to the pathogenesis of MASLD, ALD and HCC [156,190].

Pegozafermin is a FGF21 analogue with a role in the treatment of MASH and hypertriglyceridemia: “Enliven” the double-blinded randomized phase 2b trial by Loomba et al. that established, among the primary endpoints, the improvements in fibrosis of at least 1 stage without MASH worsening. This study showed encouraging results in histologically confirmed non-cirrhotic MASH (F2–F3 fibrosis), which need to be confirmed in the subsequent phase 3 trial. In the phase 2b trial, fibrosis improvement of ≥1 stage without worsening of NASH was observed in 22% of patients receiving 15 mg pegozafermin (95% CI, −9 to 38), 26% with 30 mg (95% CI, 5 to 32; *p* = 0.009), and 27% with 44 mg (95% CI, 5 to 35; *p* = 0.008), compared to 7% in the placebo group (2 of 30 patients) [191]. Promising future results may come from the ongoing phase 3 clinical trials that have the objective to assess the safety and the efficacy of two different doses of pegozafermin in the treatment both in patients with MASH-related F2–3 fibrosis and in those with MASH-related cirrhosis [192,193].

Efruxifermin is another FGF21 analogue that was tested in a phase 2 trial by Harrison et al.; a significant histological improvement of liver fibrosis and MASH resolution were detected after 24 weeks of treatment in patients with F2–3 fibrosis receiving this molecule, with satisfying tolerability. The analysis of the histological samples revealed an improvement in fibrosis (at least one stage) in 41% patients with 50 mg efruxifermin (*p* = 0.036) and 39% subjects with 28 mg efruxifermin (*p* = 0.025) versus 20% in the placebo group [194,195]. These results led to phase 3 trials to further investigate the efficacy and safety of this molecule in the treatment of F2–3 fibrosis and liver cirrhosis [196,197].

The Falcon 1 phase 2b clinical trial did not succeed in demonstrating a positive effect of pegbelfermin in the reduction of at least 1 stage of liver fibrosis, assessed histologically after 24 weeks of treatment (24–31% in pegbelfermin groups versus 14% placebo, *p* = 0.134) [198]. Alpine 2/3, a clinical trial by Harrison et al., failed to demonstrate that aldafermin could downstage liver fibrosis in patients with MASLD-related liver disease [199].

FGF21 analogues such as pegozafermin and efruxifermin were generally well tolerated in clinical trials. The most commonly reported adverse events were gastrointestinal symptoms and injection-site reactions, which were usually mild to moderate in intensity and self-limiting [191,195].

### 5.7. Bile Acids

In vitro studies by Ye et al. highlighted the possible role of ursodeoxycholic acid (largely used as a therapy in primary biliary cholangitis) in the treatment of liver fibrosis: they discovered that it can exert an effect on the HSCs, reducing collagen production and the autophagic mechanisms induced by TGF-β; the authors underline that similar results were also achieved in mouse models with induced liver fibrosis [200]. The previous study by Buko et al. documented the positive effects of both norursodeoxycholic acid and ursodeoxycholic acid on mice models, leading to significant results in terms of reversal of fibrosis especially in those treated with norursodeoxycholic that proved to be more effective in experimental settings [201].

### 5.8. Agonists of the Farnesoid X Receptor

Obeticholic acid (OCA) is an orally administered farnesoid X receptor (FXR) agonist, previously approved at a dosage of 5 and 10 mg as second-line treatment of primary biliary cholangitis [156,202] and now revoked from conditional marketing because its benefits were no longer considered to outweigh the risks of potential adverse events when compared to placebo [203]. In the Regenerate trial, Sanyal et al. documented that 25 mg OCA can determine the improvement of fibrosis of at least 1 stage in 22.4% patients, compared to 9.6% placebo (*p* < 0.0001). However, the most frequent adverse event was pruritus, particularly at higher doses, and treatment was also associated with increases in LDL cholesterol and hepatic adverse outcomes. These findings contributed to the decision to suspend its regulatory approval for NASH treatment [204].

The Tandem phase 2b trial, evaluating tropifexor (a nonsteroidal FXR agonist) alone or in combination with cenicriviroc (inhibitor of C-C chemokine receptor type 2 CCR2 and C-C chemokine receptor type 5 CCR5 receptors), did not show statistically significant antifibrotic effects [205]. These findings highlight the challenge of translating mechanistically promising compounds into meaningful clinical efficacy, emphasizing the need for robust phase 3 validation.

### 5.9. Inhibitors of the Fatty Acid Synthase

Denifanstat is an oral inhibitor of the fatty acid synthase with an activity blocking lipogenesis: for this reason, it could represent another promising tool in the treatment of liver fibrosis, because it could interrupt the progression from lipotoxicity to inflammation and fibrosis in patients with MASLD. Loomba et al. analysed its role in their multicentre, randomised, double-blind, placebo-controlled, phase 2b trial (ClinicalTrials.gov NCT04906421). Among the 1087 screened subjects, 168 were considered eligible (after liver biopsy that confirmed the presence of F2–3 fibrosis) and were subsequently treated for 52 weeks with either denifanstat (50 mg) or placebo. In the modified intention-to-treat population (mITT), 41% patients treated with denifanstat showed an improvement of fibrosis of at least one stage compared to 18% in the placebo group (*p* = 0.0102), while in the intention-to-treat population (ITT) 30% subjects in the denifanstat group achieved an improvement of fibrosis (one or more stages) compared to 14% of patients receiving placebo (*p* = 0·040). In detail, the improvement of liver fibrosis was observed in 49% of subjects with F3 fibrosis (assigned to denifanstat group) compared to 13% in the placebo group. Among diabetic patients, the reduction in liver fibrosis was obtained in 40% with denifanstat and in 19% with placebo. A group of 12 patients was taking contemporarily both denifanstat and GLP1Ra: 42% of them presented a reduction in liver fibrosis, compared to 0% in the placebo group. The encouraging results obtained by the researchers lead to the development of phase 3 trials with the aim of identifying the role of denifanstat in the treatment of MASH without fibrosis worsening and/or in the regression of fibrosis with no MASH worsening in subjects with F2–3 fibrosis [206,207]. In the phase 2b trial, it was associated with a favorable safety profile; nonetheless, patients experienced mild headache, nausea, and elevations in liver enzymes [207].

### 5.10. Inhibitors of the Renin–Angiotensin System

Some experimental attempts were made to evaluate the role of the inhibition of the renin-angiotensin system (particularly losartan) in the treatment of liver fibrosis, with promising results in small studies involving patients with MASLD-related liver fibrosis [208] or in a group of liver-transplanted patients with post-transplant risk of recurrence of HCV-related liver disease (even if the risk of this complication has been widely reduced nowadays by the introduction of direct acting antivirals that avoid the recurrence of HCV infection on the transplanted liver) [209]. Gu et al., through a mouse model of MASLD-related HCC, highlighted a potentially important role of losartan as an anti-neoplastic adjuvant treatment due to its capacity to enhance the invasion of tumor tissue by CD8 T cells, normally inhibited by peri-lesional fibrosis, and to inhibit the TGF-β signalling with subsequent deposition of collagen and reducing fibroblasts with immunosuppressive effect, thus potentiating the effect of immune checkpoint inhibitors used for HCC treatment [210].

Angiotensin inhibitors were also investigated with the purpose of finding an anti-fibrotic treatment. Okanoue et al. documented no significant results (*p* = 0.203) applying apararenone (nonsteroidal antimineralocorticoid) for the decrease in more than one-stage fibrosis (assessed through non-invasive tests) [211].

### 5.11. Other Medications

Among the medications that were examined on their potential effect in improving liver fibrosis, there are omega-3 polyunsaturated fatty acids without significant results [212,213], except for icosabutate that showed the property of decreasing inflammation, with a beneficial effect on hepatic fibrosis in mice [214].

Further investigations are needed to confirm in humans the results obtained in mice with MASLD-related liver disease by aramchol, a partial inhibitor of hepatic stearoyl-CoA desaturase (SCD1) [215].

No significant data are available regarding lipid-soluble vitamin E, despite its role as antioxidant and anti-inflammatory agent, and no phase 3 trial were performed so far [216].

Chen et al. investigated in a mouse-model the anti-fibrotic effect of inteleukin-10 (IL-10) through the stimulation of the function of NK cells (involving their activation, migration and cytotoxic function), thus reducing the progression of the fibrosis in liver, but further in vivo research is required to fully comprehend this complex interplay that undoubtedly involves also HSCs [217].

Despite previously investigated as a potential treatment for liver fibrosis due to its anti-inflammatory effects, Naffaa et al. recently discovered that long-term use of colchicine is linked to incident cirrhosis; for this reason, its use for long periods should be reserved to those patients who do not have alternative treatments for their disease (such as Familial Mediterranean Fever) [218].

No significant results were found in terms of histological decrease in liver fibrosis in studies using silymarin [219].

Some researchers also investigated in small groups of patients the role of statins in the reduction in liver fibrosis in patients with MASLD and in the lowering of the risk of HCC, with significant results [220,221], but larger clinical trials are needed to confirm this relevant evidence.

Another drug that has been investigated for its possible effect on liver fibrosis is pioglitazone that belongs to the family of thiazolidinediones, activating the receptor of peroxisome proliferator-activated (PPAR)γ: some molecules included in this group were withdrawn in several nations due to enhanced incidence of drug-mediated hepatitis (troglitazone), raise in incidence of bladder cancer (pioglitazone), cardiovascular events (rosiglitazone). Despite being able to improve steatohepatitis at the examination of histological samples of the liver, pioglitazone was not clearly proven to determine a regression of the fibrosis after long term treatment [222]. However, due to the harmful adverse events related to this pharmacological class of molecules, no wide phase 3 trials were conducted, and pioglitazone is no longer available in several European countries.

Table 2 summarizes the main pharmacological agents investigated for MASH-related liver fibrosis.

### 5.12. A Panoramic Overview on Anti-Fibrotic Medications

**Figure 2:**
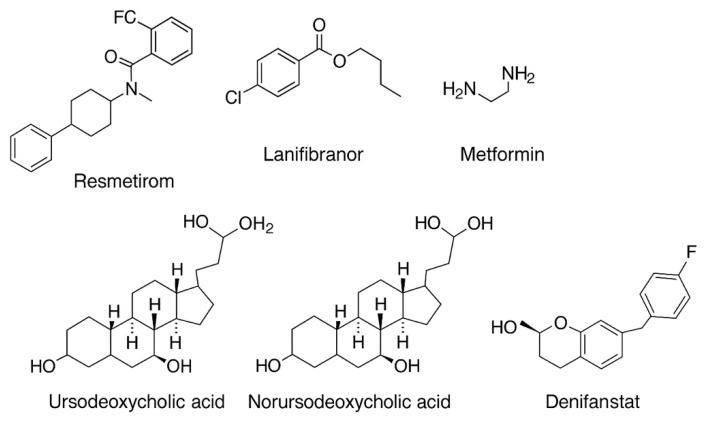
Chemical structure of selected antifibrotic agents discussed *. * Biologic agents and peptides (e.g., GLP-1 receptor agonists and FGF21 analogs) are not represented due to their large molecular size and structural complexity, which limit their inclusion in standard chemical structure formats.

Souza et al. recently conducted a systematic review and network meta-analysis including 29 randomized controlled trials and a total of 9324 patients with metabolic dysfunction-associated steatohepatitis (MASH), aiming to compare the efficacy of pharmacological agents for fibrosis regression. Using surface under the cumulative ranking curve (SUCRA) analysis, the authors established a probabilistic ranking of available therapies. Pegozafermin demonstrated the highest likelihood of inducing fibrosis regression (SUCRA 79.92), suggesting it may be the most effective monotherapy currently under investigation. This was followed by combination regimens, particularly cilofexor plus firsocostat (SUCRA 71.38) and cilofexor plus selonsertib (SUCRA 69.11), indicating that dual pathway modulation may offer additive or synergistic benefits. Denifanstat (SUCRA 60.64), a fatty acid synthase inhibitor, also showed promising results. Conversely, therapies such as obeticolic acid, tirzepatide, vitamin E (alone or in combination with pioglitazone), resmetirom, semaglutide, and lanifibranor achieved lower SUCRA scores (ranging from 58.86 to 46.56), suggesting more modest or inconsistent effects on fibrosis regression in this patient population. These findings underscore the potential superiority of newer agents such as pegozafermin and combination regimens targeting multiple pathogenic pathways. However, direct head-to-head comparisons remain limited, and further phase III studies are necessary to confirm these rankings and to assess long-term outcomes and safety profiles [223]. Figure 2 describes the chemical structure of selected antifibrotic agents discussed.

## 6. Microbiota-Targeted Therapies in Liver Fibrosis

In recent years, a vivid interest bloomed in developing therapeutic strategies that can interfere with this double directional interaction, such as the administration of probiotics, prebiotics, symbiotics, antibiotics, and fecal microbiome transplantation (FMT).

Probiotics are widely administered with the purpose of re-establishing a healthy gut microbiome (for example, after infectious diseases involving the intestine), because they include living microorganisms (bacteria and yeast) that have the propriety to restore the epithelial barrier of the intestine and modulate its function [224].

In a group of MASH-model rats Sawada et al. noticed that the concomitant administration of probiotics and angiotensin-II type 1 receptor blockers (ARBs) determined the suppression of fibrogenic processes through both the reduction in the permeability of the intestinal epithelial barrier (by probiotics) and the direct inhibition of HSCs (by ARBs) [225]. A double-blind randomized clinical trial by Alisi et al., on a population of 44 obese children with histologically diagnosed MASLD, highlighted the role of the probiotic mixture VSL#3 (administered once per day for four months) in significantly reducing the BMI and the severity of fatty liver disease: they hypothesized that this result may derive from an increase in circulating levels of GLP-1 provoked by VSL#3, mimicking a mechanism induced by nutrients absorption, with consequent insulin release and glucagon inhibition, delay of the emptying of the stomach and enhancement of the loss of weight with positive effects on the metabolic general balance [226]. Wong et al. proved the role of “Lepicol” a combination of probiotics (*Lactobacillus delbrueckii*, *Lactobacillus acidophilus*, *Lactobacillus plantarum*, *Bifidobacterium bifidum* and *Lactobacillus rhamnosus*) in the reduction in liver inflammation and fat levels, after 6 months of treatment, in a study involving 20 patients with MASH diagnosed through biopsy [227].

Although these studies imply that probiotics may play an important role in the treatment of liver fibrosis (mainly interfering with its pathogenetic mechanisms, interrupting or reducing chronic inflammation), a small trial by Solga et al. on four adult subjects reported opposite and controversial results [228]. For this reason, larger studies are mandatory to deepen the knowledge regarding the effects of probiotics on inflammation and its potential role in the treatment of liver fibrosis.

Alongside probiotics, prebiotics have also been investigated. They are indigestible oligosaccharides (e.g., inulin and lactulose) that have positive effects reducing intestinal permeability and favouring the expansion of certain strains of beneficial bacteria, as stated in a double-blind study on 30 obese women whose diet was enriched with inulin-type fructans, obtaining a selective increase in *Faecalibacterium prausnitzii* and *Bifidobacterium*, a decrease in LPS levels, *Bacteroides*, and *Propionibacterium*, leading to a reduction in fat mass [229,230].

Symbiotics are a mixture of prebiotics and probiotics that can activate the selective increase in specific beneficial bacterial strains, the reduction in pro-inflammatory factors and consequently MASH activity index, but only an exiguous amount of data is available regarding their potential direct effect on liver fibrosis [231,232].

Despite their role in reducing liver inflammation, there is still no clear evidence about their possible involvement in inhibiting liver fibrosis.

Antibiotics represent another important tool for the modulation of gut microbiota: rifaximin is a non-absorbable antibiotic, already studied in several trials regarding patients with liver diseases. There is scientific evidence that rifaximin can suppress the progression of fibrosis, particularly reducing the inflammatory burden and the activation of HSCs, with important repercussions specifically on metabolic- or alcohol-related liver damage, but also in case of iron overload in the liver [233,234,235]. Encouraging results came from a phase 2 double-blind trial by Israelsen et al. on 136 patients with alcohol-related liver disease, that hypothesized that Rifaximin-α could block the progression of liver fibrosis through the reduction in the inflammatory cascade; however, due to the scarce statistical significance of their results, further investigation is needed [236].

Fecal microbiota transplantation (FMT) is a current topic in several medical fields due to the contribution given by gut dysbiosis in the etiology of different diseases: there is a constantly growing interest in FMT and its possible implications in the treatment of MASLD- and alcoholic-related liver fibrosis and its complications, through the restoration of the epithelial barrier of the intestine [237].

A double-blind trial by Ng et al. recently remarked that reiterated FMTs associated with lifestyle changes in patients with obesity and type 2 diabetes have a significant impact on the engraftment of microbiota (*p* < 0.05) and on the improvement of liver stiffness (*p* < 0.05) compared to FMT alone and lifestyle changes alone [238].

Oguri et al. investigated the composition of gut microbiota in murine models with induced liver fibrosis/cirrhosis treated with rifaximin. They observed that the subjects with an improved of fibrosis and hyperammonemia presented an increase in a commensal strain of *Akkermansia muciniphila*. For this reason, the authors observed that the livers of mice in the group supplemented with this bacterium presented a significant smaller area of fibrosis (*p* = 0.0012). This evidence hints at a potential future primary role of the supplementation of *A. muciniphila* in the improvement of liver fibrosis [239]. Moreover, a higher abundance of *Sellimonas* and a lower abundance of *Ruminococcaceae UCG 013* and *Ruminoclostridium* genera was observed with liver fibrosis, alongside with other predictors of MASLD severity, suggesting their role in the prediction of MASLD worsening [240]. Table 3 summarizes the main microbiota-based therapies.

## 7. Conclusions and Future Directions

Liver fibrosis derives from progressive liver damage and can lead to severe complications, including cirrhosis, hepatic failure, and hepatocellular carcinoma. Therefore, it is crucial to identify therapies capable of effectively interrupting inflammatory processes and fibrosis development, potentially tailored to distinct etiologies. Despite promising results in preclinical models, these findings are not fully reproducible in humans, likely due to biological differences. Consequently, the primary anti-fibrotic strategy remains the removal of the causal agent when possible, although this generally stabilizes existing damage rather than reversing it. Liver transplantation remains the only definitive solution but is not universally applicable and carries significant implications for quality of life and long-term outcomes. Current pharmacological options face several limitations, including incomplete fibrosis regression, variable patient responses, adverse effects, and limited long-term safety data. Additionally, the number of clinical trials remains limited, often involving small populations without head-to-head comparisons. To address these gaps, future studies should adopt innovative trial designs, such as adaptive platform trials, which enable simultaneous evaluation of multiple agents, dropping ineffective arms, and introducing new treatments based on emerging data. Lifestyle modifications must also be emphasized as a foundational strategy to prevent fibrosis progression to cirrhosis or carcinoma. Weight management, dietary interventions, regular physical activity, and avoidance of alcohol and hepatotoxic substances are essential measures that can complement pharmacological and microbiota-based therapies in mitigating disease progression. The combination of agents targeting different fibrogenic pathways may enhance therapeutic efficacy, but comprehensive safety evaluations are essential. Moreover, it is critical to determine whether histological regression translates into meaningful reductions in clinical complications, such as portal hypertension and hepatic decompensation. Another major challenge is the invasive nature of liver fibrosis assessment; reliance on liver biopsy limits patient enrollment and increases risk. Development and validation of non-invasive biomarkers and imaging techniques should be prioritized to facilitate trial design, improve recruitment, and enhance patient adherence. Finally, alongside established and emerging pharmacological strategies, future research should explore novel approaches—including immunotherapy, gene editing, and modulation of the gut–liver axis—to disrupt chronic inflammation and halt fibrogenesis at its root. Well-powered, long-term trials are required to confirm efficacy, durability, and safety across diverse patient populations, ultimately aiming to achieve not only stabilization but also possible reversal of liver fibrosis.

## Figures and Tables

**Table 1:** Genetic and epigenetic mechanisms at the basis of liver fibrosis.

Mechanism	Key Points
Genetic Factors	PNPLA3 (rs738409 C>G): Impaired lipid hydrolysis → HSC activation → ECM deposition. TM6SF2 (rs58542926 C>T): Disrupted VLDL secretion → Lipid accumulation → Steatosis & fibrosis. MBOAT7 (rs641738): Increased fibrosis risk in chronic liver diseases.
DNA Methylation	Hypermethylation: Silences anti-fibrotic genes (e.g., PPARγ, SOCS1), preventing fibrosis resolution. Hypomethylation: Activates pro-fibrotic genes (e.g., TGFβ1), promoting fibrosis.
Epigenetic Regulators	EZH2: Gene silencing → HSC activation. HDACs/SIRT1: Histone acetylation → Drives fibrosis. KDM6B: Removes repressive marks → Activates fibrogenic genes.
Non-Coding RNAs	miR-21: Promotes HSC activation. miR-29: Downregulated → Excessive ECM accumulation. miR-122: Downregulated → Link to fibrosis and HCC. H19/MEG3/MALAT1: Regulate chromatin and fibrosis pathways.

Abbreviations: ECM, Extracellular matrix; EZH2, enhancer of zeste homolog 2; HDACs, histone deacetylases; HSC, hepatic stellate cells; MALAT1, metastasis associated lung adenocarcinoma transcript 1; MBOAT7, membrane-bound O-acyltransferase domain-containing protein 7; MEG3, maternally expressed 3; miR, microRNAs; PNPLA3, patatin-like phospholipase domain-containing 3; PPARγ, peroxisome proliferator-activated receptor gamma; SIRT 1, sirtuin 1; SOCS1, suppressor of cytokine signaling 1; TGFβ1, transforming growth factor beta 1; VLDL, very-low-density lipoproteins.

**Table 2:** Overview of investigational and approved drugs for MASH-related liver fibrosis.

Drug	Drug Type	Mechanism of Action	Key Clinical Trial and Population	Main Outcomes
Resmetirom [157,158,159,160,161]	Liver-directed THR agonist	THR-β agonist; reduces hepatic lipogenesis and TGF-β activity	MAESTRO-NASH, Phase III, non-cirrhotic MASH F2–F3	Reduced inflammation, slower fibrosis progression; AEs: nausea 22%, diarrhea 33%, vomiting 11%
Semaglutide [162,163,164,165,166]	GLP-1 RA	Mimics GLP-1 → glycemic control, appetite/weight reduction	ESSENCE, Phase III, MASH patients	MASH resolution 62.9% vs. 34.1%; fibrosis improvement 37% vs. 22.5%; GI AEs common
Tirzepatide [169]	Dual GLP-1/GIP RA	Dual GLP-1 and GIP agonist	Phase II, dose-finding, MASH	≥1-stage fibrosis improvement 51–55% vs. 30% placebo; GI AEs dose-related
Cotadutide [170]	Dual GLP-1/Glucagon RA	Dual GLP-1/glucagon receptor agonist	Phase II, MASH	Reduction in fibrosis scores (FIB-4/NFS, *p* = 0.010)
Liraglutide [170]	GLP-1 RA	GLP-1 receptor agonist	Various trials in MASH	No significant fibrosis improvement; well tolerated
Survodutide [171]	Dual GLP-1/Glucagon RA	Dual GLP-1/glucagon receptor agonist	Phase II, MASH	≥1-stage fibrosis reduction: 34–36% vs. 22% placebo; study not powered for fibrosis
Dapagliflozin/Empagliflozin/Licogliflozin [156,172,173,174,175]	SGLT2 inhibitors	Block renal glucose reabsorption; modulate HSC-activating microRNAs	Small studies & large cohort analyses, T2DM	Potential fibrosis reduction in advanced fibrosis; lower risk of cirrhosis vs. DPP-4 inhibitors; effects may be weight-mediated
Lanifibranor [176,177,178,179,180]	Pan-PPAR agonist	Activates PPAR-α, -β/δ, -γ → modulates lipid metabolism, inflammation, fibrogenesis	Phase II, MASH	≥1-stage fibrosis improvement 48% (1200 mg), 34% (800 mg), vs. 29% placebo; AEs: weight gain, edema
Metformin [156,181,182,183,184,185,186,187,188,189]	Biguanide	Glucose-lowering, anti-inflammatory, anti-fibrotic	Observational/animal studies, MASLD/T2DM	>50% regression in fibrosis; inhibits TGF-β, SMAD3, HSC activation; improves transplant-free survival; reduces HCC risk
Pegozafermin [191,192,193,223]	FGF21 analogue	Hormone-like factor → reduces lipogenesis, increases insulin sensitivity	Phase 2b, non-cirrhotic MASH F2–F3	≥1-stage fibrosis improvement 22–27% vs. 7% placebo; well tolerated; phase 3 ongoing
Efruxifermin [194,195,196,197]	FGF21 analogue	Same as above	Phase 2, MASH F2–F3	≥1-stage fibrosis improvement 39–41% vs. 20% placebo; MASH resolution; good tolerability
Pegbelfermin/Aldafermin [198,199]	FGF21 analogues	Same	Phase 2b, MASH	Failed to show significant fibrosis improvement
Obeticholic acid (OCA) [156,202,203,204]	FXR agonist	Farnesoid X receptor agonist → modulates bile acid metabolism, inflammation, fibrosis	Regenerate, Phase III, MASH	≥1-stage fibrosis improvement 22.4% vs. 9.6%; AEs: pruritus, LDL ↑; approval withdrawn for NASH
Tropifexor ± Cenicriviroc [205]	FXR agonist ± CCR2/5 inhibitor	Same	Phase 2b	No significant antifibrotic effect
Denifanstat [206,207]	Fatty acid synthase inhibitor	Blocks lipogenesis → prevents lipotoxicity-driven fibrosis	Phase 2b, F2–F3 fibrosis	≥1-stage fibrosis improvement 41% mITT vs. 18% placebo; mild AEs; phase 3 ongoing
Losartan/Apararenone [208,209,210,211]	Renin-angiotensin system inhibitors	Inhibit RAAS; reduce TGF-β signaling and fibroblast activation	Small clinical studies, MASLD/HCC	Experimental antifibrotic effects; results inconsistent or non-significant
Pioglitazone/Thiazolidinediones [222]	PPARγ agonist	Improves steatohepatitis histologically	Various trials	No clear long-term fibrosis regression; adverse events limit use
Vitamin E (±Pioglitazone) [223]	Antioxidant/anti-inflammatory	Reduce oxidative stress and inflammation	Various trials	Modest/inconsistent effect on fibrosis
Statins [220,221]	HMG-CoA reductase inhibitors	Lipid-lowering; potential anti-inflammatory	Small clinical studies	Possible fibrosis reduction and lower HCC risk; larger trials needed
Icosabutate/Omega-3 PUFA [212,213,214]	Fatty acids	Anti-inflammatory	Preclinical/small trials	Icosabutate reduces inflammation and improves fibrosis in mice; omega-3 PUFA no significant effect
Aramchol [215]	SCD1 partial inhibitor	Reduces lipogenesis	Preclinical	Potential fibrosis benefit in mice; no human phase 3 data
IL-10 [217]	Immunomodulator	Activates NK cells → antifibrotic	Preclinical	Reduces fibrosis in mice; requires further in vivo research
Colchicine [218]	Anti-inflammatory	Inhibits microtubule polymerization	Observational	Long-term use linked to cirrhosis; limited clinical use
Silymarin [219]	Antioxidant/anti-inflammatory	Free radical scavenger	Various small studies	No significant histological improvement in fibrosis

**Table 3:** Microbiota-based therapies for MASLD and liver fibrosis: mechanisms and main outcomes.

Therapy	Type	Mechanism of Action	Main Outcomes
Probiotics (VSL#3, Lepicol, others) [224,225,226,227]	Live microorganisms	Restore intestinal epithelial barrier, modulate gut microbiome, reduce intestinal permeability, anti-inflammatory	Reduced BMI and fatty liver severity; reduced liver inflammation and fat levels; variable results across studies; small trials
Prebiotics (inulin, lactulose) [230]	Indigestible oligosaccharides	Promote beneficial bacteria growth, reduce intestinal permeability	Increased Faecalibacterium prausnitzii and Bifidobacterium; decreased LPS, Bacteroides and Propionibacterium; reduced fat mass
Symbiotics [231,232]	Combination of prebiotics and probiotics	Selective growth of beneficial bacteria, reduce pro-inflammatory factors	Potential reduction in MASH activity index; direct effect on liver fibrosis not well established
Antibiotics (Rifaximin) [233,234,235,236]	Non-absorbable antibiotic	Modulate gut microbiota, reduce inflammation, inhibit HSC activation	Potential suppression of fibrosis progression, reduced inflammatory burden; further studies needed due to limited statistical significance
Fecal Microbiota Transplantation (FMT) [237,238]	Microbiome transfer	Restore intestinal barrier, modulate gut microbiota	Reiterated FMT plus lifestyle changes improved microbiota engraftment and liver stiffness compared to FMT alone or lifestyle alone
Akkermansia muciniphila supplementation [239]	Commensal bacteria	Reduce fibrosis, improve hyperammonemia	Significant reduction in fibrosis area (*p* = 0.0012)
Gut microbiota composition markers [240]	Microbiome profiling	Predict MASLD severity and fibrosis progression	Higher abundance of Sellimonas and lower Ruminococcaceae UCG 013/Ruminoclostridium linked with fibrosis and MASLD progression

## Data Availability

Figures were created with BioRender software (https://biorender.com/ (accessed on 29 April 2025).

## Abbreviations

The following abbreviations are used in this manuscript:

ACLD	Advanced chronic liver disease
AIH	Autoimmune hepatitis
ARBs	Angiotensin-II type 1 receptor blockers
ASK1	Apoptosis Signal-regulating Kinase 1
Bambi	BMP and activin membrane-bound inhibitor
BDL	Bile duct ligation
CCl_4_	Carbon tetrachloride
DAMPs	Damage-associated molecular patterns
DPP	Dipeptideyl peptidase 4
ECM	Extracellular matrix
EZH2	Enhancer of zeste homolog 2
FFAs	Free fatty acids
FGF21	Fibroblast Growth Factor 21
FIB-4	Fibrosis-4
FMT	Faecal microbiome transplantation
FXR	Farnesoid X receptor
GLP-1	Glucagon-like peptide 1
GLP1RA	Glucagon-like peptide-1 receptor agonists
GLS1	Glutaminase 1
HBV	Hepatitis B virus
HCC	Hepatocellular carcinoma
HCV	Hepatitis C virus
HDACs	Histone deacetylases
HDV	Hepatitis D virus
HK2	Hexokinase 2
HMGB1	High-mobility group box 1
HSCs	Hepatic stellate cells
IFN-γ	Interferon-gamma
IL-1β	Interleukin-1 beta
IL-10	Inteleukin-10
ITT	Intention-to-treat population
KCs	Kupffer cells
KDM6B	Lysine demethylase 6B
kPa	Kilopascals
LDHA	Lactate dehydrogenase A
lncRNAs	Long non-coding RNAs
LPS	Lipopolysaccharide
MALAT1	Metastasis Associated Lung Adenocarcinoma Transcript 1
MASH	metabolic dysfunction-associated steatohepatitis
MASLD	Metabolic dysfunction-associated steatotic liver disease
MEG3	Maternally Expressed 3
miRNAs	MicroRNAs
mITT	Modified intention-to-treat population
ML NASH CRN	Machine Learning NASH Clinical Research Network
MRE	Magnetic resonance elastography
NASH	Non-alcoholic fatty liver disease
NF-κB	Nuclear factor kappa B
NFS	NAFLD fibrosis score
NK	Natural Killer
NKT	Natural Killer T
NOX	NADPH oxidase
OCA	Obeticholic acid
PAMPS	Pathogen Associated Molecular Patterns
PBC	Primary biliary cholangitis
PDGF	Platelet-derived growth factor
PKM2	Pyruvate kinase M2
PNPLA3	Patatin-like phospholipase domain-containing 3
PPAR	Peroxisome proliferator-activated receptor
PRRs	Pattern recognition receptors
PSC	Primary sclerosing cholangitis
ROS	Reactive oxygen species
SAF	Steatosis Activity Fibrosis
SCD1	Stearoyl-CoA desaturase
SCFAs	Short-chain fatty acids
SGLT-2	Sodium–glucose cotransporter 2
SIRT1	Sirtuin 1
SUCRA	Surface under the cumulative ranking curve
SWE	Shear wave elastography
TCA	Tricarboxylic acid
TE	Transient elastography
TGFβ1	Transforming growth factor beta 1
THR	Thyroid hormone receptor agonists
TLRs	Toll-like receptors
TM6SF2	Transmembrane 6 superfamily member 2
TNF-α	Tumor necrosis factor alpha
VLDL	Very-low-density lipoproteins
